# Face masks are less effective than sunglasses in masking face identity

**DOI:** 10.1038/s41598-023-31321-4

**Published:** 2023-03-15

**Authors:** Charles C.-F. Or, Kester Y. J. Ng, Yiik Chia, Jing Han Koh, Denise Y. Lim, Alan L. F. Lee

**Affiliations:** 1 Division of Psychology, School of Social Sciences, Nanyang Technological University, 48 Nanyang Avenue, Singapore, 639818 Singapore; 2 Department of Psychology, Lingnan University, Tuen Mun, Hong Kong

**Keywords:** Human behaviour, Object vision

## Abstract

The effect of covering faces on face identification is recently garnering interest amid the COVID-19 pandemic. Here, we investigated how face identification performance was affected by two types of face disguise: sunglasses and face masks. Observers studied a series of faces; then judged whether a series of test faces, comprising studied and novel faces, had been studied before or not. Face stimuli were presented either without coverings (full faces), wearing sunglasses covering the upper region (eyes, eyebrows), or wearing surgical masks covering the lower region (nose, mouth, chin). We found that sunglasses led to larger reductions in sensitivity (*d’*) to face identity than face masks did, while both disguises increased the tendency to report faces as studied before, a bias that was absent for full faces. In addition, faces disguised during either study or test only (i.e. study disguised faces, test with full faces; and vice versa) led to further reductions in sensitivity from both studying and testing with disguised faces, suggesting that congruence between study and test is crucial for memory retrieval. These findings implied that the upper region of the face, including the eye-region features, is more diagnostic for holistic face-identity processing than the lower face region.

## Introduction

The COVID-19 pandemic has made facial occlusions more prevalent due to mask-wearing, which covers multiple lower facial features including the entire mouth, the lower part of the nose, and the lower face outline. This has renewed interest in how facial occlusions might affect face identification, as the amount of information available in identifying faces is reduced.

Since the pandemic, several studies have demonstrated that occlusion by face mask leads to general impairment to face identification. For example, Freud et al.^1,2^ suggested that presenting masked faces during encoding and/or retrieval stages in online Cambridge Face Memory Tests (CFMT) persistently led to poorer scores than presenting only unoccluded faces in both stages. Such impairment was also observed in another in-person face learning-and-recognition task by Hsiao et al.^3^. Marini et al.^4^ demonstrated that unoccluded faces were re-identified more accurately when unoccluded faces, rather than masked faces, were encoded. When matching two simultaneously presented faces^5,6^, sensitivity (*d’*) decreased when one or both faces were masked, while response bias varied across experiments.

Pre-COVID-19 studies predicted more limited, or even an absence of, impairment to face identification, despite their focus on occluding single lower features (e.g. occluding the mouth alone). Rather than a general occlusion effect, McKelvie^7^ demonstrated that face memory worsened only when presenting different (i.e. incongruent) face types, specifically, mouth-occluded faces during encoding and full faces during retrieval (and vice versa), but presenting the same (i.e. congruent) mouth occlusions in both stages did not impair face memory compared to congruently presenting full faces in both stages. The impairment, which was limited to incongruent conditions, may support the encoding specificity principle, where stimuli presented under congruent conditions across stages would lead to superior performance^8,9^. Encoding specificity has been demonstrated in aspects of face memory research (e.g. removing external facial areas^10^; changing lighting^11^), limiting generalization of encoded facial information to novel viewing conditions. Nevertheless, even under incongruent conditions, masking the nose or mouth alone might not impair face memory, in the case of familiarity judgments of masked celebrity faces that were expected to be encoded previously as unoccluded faces^12^.

Other studies focused on the effect of exposing only a single feature on face identification. For example, Manley et al.^13^ studied memory for faces covered by ski masks, leaving only the eyes visible in lineup experiments. They found that congruently masking faces led to comparable sensitivity and response bias with congruently presenting full faces, but incongruently masking faces resulted in a decline in sensitivity or a change in response bias, again in agreement with encoding specificity. Although these pre-COVID-19 studies did not have lower facial features occluded in the same way as using face masks, they demonstrate the possibility that occlusion of lower facial features may not always deteriorate face identification performance, especially under congruent conditions.

In contrast, findings from multiple studies suggest that occlusion of the upper region of the face (including the eyes) impairs face identification, consistent with the notion that the eyes provide crucial diagnostic information for face identity processing (e.g.^14,15^). McKelvie^7^ suggested that masking the eyes in one or both memory test stages led to poorer face memory than congruently presenting full faces. Masking the eyes of celebrity faces was also detrimental to familiarity judgments^12,16^. Using sunglasses for more natural occlusions, Graham and Ritchie^17^ found lower sensitivities in matching two faces either or both with sunglasses than in matching two full faces. When videos of faces with or without sunglasses were encoded prior to retrieval with lineups of full faces only, Mansour et al.^18^ showed that encoding faces with sunglasses reduced sensitivity from encoding full faces. However, it should be noted that Hockley et al.^19^ only found reduced sensitivity with incongruent memory test stages, while congruently presenting faces with sunglasses resulted in little decline in sensitivity from congruently presenting full faces. Thus, they attributed the effects to encoding specificity rather than a general effect from eye occlusion.

To date, few studies directly compared the effects of occluding the upper and the lower regions of the face on face identification. While earlier studies (e.g.^7,12^) compared the effects of occluding single features of the face and suggested the superiority of the eyes to the nose or mouth in face identification performance, more recent studies compared the effects of more natural occlusions. For example, Nguyen and Pezdek^20^ conducted a face memory experiment about encoding full faces or disguised faces (sunglasses covering the upper region, or bandanas covering the lower region), followed by retrieval with full faces only. They found that sunglasses consistently lowered sensitivity, while bandanas led to decline in sensitivity only for black observers but not white observers. In contrast, Noyes et al.^21^ suggested that, when compared to matching two full faces, matching a face wearing a face mask with a full face generally led to a larger decrease in accuracy than matching a face wearing sunglasses with a full face. Using similar face matching tasks, however, Bennetts et al.^22^ found similar decreases in sensitivity and similar increases in bias when comparing the effects of sunglasses and surgical masks relative to full faces. Thus, the degree to which the lower or upper occlusion separately affects face identification remains unclear, especially under different congruent and incongruent encoding/retrieval conditions which were not systematically investigated in the aforementioned studies.

In this study, we sought to directly compare the effects of occluding the upper and the lower regions of the face in three face memory experiments. In Experiment 1, observers performed three face memory tasks (Condition 1: uncovered full-faces, Condition 2: faces with sunglasses, Condition 3: faces with surgical masks), with disguise types congruent across encoding and retrieval stages. Experiments 2–3 were similar to Experiment 1, but introduced incongruence in disguise type between encoding and retrieval (Experiment 2: learning disguised faces, testing with full faces; Experiment 3: learning full faces, testing with disguised faces). These three experiments would allow for additional comparisons among different congruent and incongruent disguise scenarios, which to our knowledge were not systematically contrasted to test for encoding specificity. Together, the present study allowed us to understand the relative contributions of the upper and lower face regions to face identity processing, in conjunction with the effects of congruent and incongruent face disguise types across memory test stages.

## General methods

### Participants

Singaporean Chinese observers experienced in recognizing Chinese faces, but unaware of the purpose of study and unfamiliar with the face stimuli, were recruited. Their visual acuities were either normal or corrected to normal. All observers provided written informed consent and were remunerated. The Psychology Ethics Committee of Nanyang Technological University approved of the study in accordance with the Declaration of Helsinki.

The minimum effect size was set to be *δ* = 0.60 based on previous studies^7,20,21^ comparing the effects between occluding the upper and lower parts of the face. At a power of 0.80 and an alpha of 0.05, the minimum required sample size would be *N* = 24, which was used as our sample size in each experiment.

### Stimulus display

All stimuli were displayed in a dimly lit room on a BenQ XL2420Z monitor (refresh rate: 120 Hz, colour depth: 24 bits/pixel, screen resolution: 1920 × 1080 pixels, viewing distance: 70 cm, pixel size: 0.023°) using Psychopy 3.2.4 for Windows installed in a Dell XPS Desktop computer with a NVIDIA GeForce GTX 745 graphics card. The screen luminance ranged from 0.09–23.61 cd/m^2^.

### Stimuli

**Figure 1:**
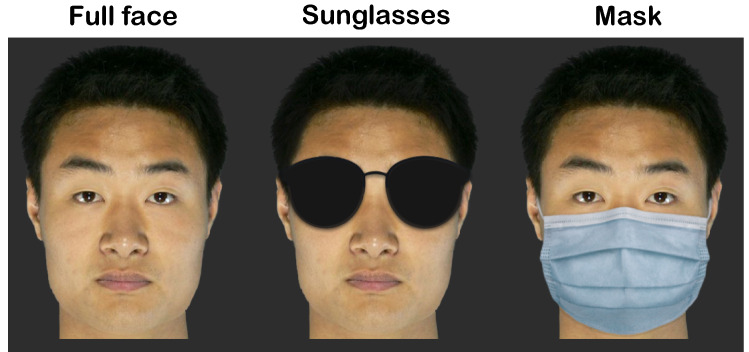
Example faces of the same identity in the 3 disguise types. All faces were displayed against a black screen, with clearly distinguishable head outlines.

Colour face images of 130 male Chinese individuals were downloaded from the Oriental Face Database owned by the Institute of Artificial Intelligence and Robotics of Xi’an Jiaotong University (http://gr.xjtu.edu.cn/web/jianyi/english-version) with permission. These individuals were photographed in full-front views and having neutral expressions. Figure 1(left) shows one of the face images, with informed consent obtained by the database owner to publish the image in an online open-access publication. After rescaling, the head size subtended a standardized height of 9.61° (crown to chin) and a width ranging from 6.60° to 7.94° (mean: 7.22°). Throughout the experiments, the head size was jittered randomly (80–120% of the original size) for each face presentation during learning and memory test sessions. This was designed to reduce the possibility of using low-level image features to identify faces (see “Discussion”). Distinctive facial features, such as blemishes, were removed for being potential identification cues. The face images were displayed against a uniform black background, showing clearly distinguishable head outlines.

Each full face image was digitally modified to generate the disguised versions (Fig. 1, middle and right). For the sunglasses version, black sunglasses were added to cover both eyes and eyebrows. For the mask version, a light blue surgical mask was added to cover the lower part of the face just below the eyes, including the chin, the mouth and most of the nose. The shapes of the sunglasses and masks were identical across faces. In total, our database contained 390 face images (130 identities × 3 disguise types).

### Procedure

In each experiment, there were three conditions, each started with learning a series of faces followed by a face memory test. The orders of three conditions were counterbalanced across observers.

#### Face learning

In each condition, observers started with remembering 21 faces (all with the same disguise type) by passive viewing. They were told that such faces would reappear in the memory test among other new faces. The 21 faces (all distinct individuals) were randomly presented in sequence, and then repeated immediately in a second sequence in a different random order. Together, the face learning session consisted of 42 successive face presentations (21 faces × 2 repetitions). Prior to each face presentation, a grey fixation cross (size: 0.36° × 0.36°; duration: 500 ms) appeared centrally against a uniform black screen. Immediately after the fixation cross disappeared, a face was presented centrally for 2000 ms. Accordingly, each face was presented for 4000 ms in total across two repetitions. Pilot experiments have verified that such setups optimized face learning.

#### Face memory test

Immediately following face learning, observers performed a face memory test containing 21 faces they just learnt (“studied faces”), plus 21 new faces (“distractor faces”). These 42 test faces (all with the same disguise type) were each presented only once for 350 ms in a randomized sequence (i.e. 42 trials) at the centre of screen. During face presentations, observers were required to maintain central fixation with the help of a preceding central fixation cross (duration: 500 ms, as in face learning). After each face vanished, the observer’s task was to indicate whether the face had been studied before or not, by pressing one of two keys. No feedback was given for any responses. Following the key press, the next trial started automatically.

After completing a condition, a 3-min break was provided before the next condition commenced. Within an experiment (approximate duration: 20 min), the 42 face identities were different across the three conditions (our database contained 130 distinct face identities; see “Stimuli”).

### Data analysis

The face memory for each observer was analysed using sensitivity *d’*:

1 $$d^{\prime} = {\text{ z}}\left( {\text{H}} \right) \, {-}{\text{ z}}\left( {\text{F}} \right),$$

where H is the hit rate, i.e. proportion of studied faces identified correctly in the memory test, and F is the false alarm rate, i.e. proportion of distractor faces identified incorrectly as having studied during face learning. Where *d’* would become an infinite number, H = 1 was converted to H = 1 – 1/(2*n*_*s*_), *n*_*s*_ = 21 (number of trials presenting studied faces), and F = 0 to F = 1/(2*n*_*d*_), *n*_*d*_ = 21 (number of trials presenting distractor faces)^23^.

Response bias was measured by criterion *c*:

2 $$c = \, {-}\left[ {{\text{z}}\left( {\text{H}} \right) \, + {\text{ z}}\left( {\text{F}} \right)} \right]/{2}{\text{.}}$$

A negative value of *c* represents a liberal bias (i.e. a tendency to label test faces as having studied), whereas a positive value of *c* represents a conservative bias (i.e. a tendency to label test faces as novel).

For each experiment, the hit rates, false alarm rates, sensitivities *d’* and criteria *c* were separately analysed using one-way repeated measures ANOVAs to compare the results across conditions. Wherever applicable, Holm-Bonferroni corrections were applied in within-subjects post hoc pairwise comparisons.

## Experiment 1: congruent learning and testing of faces

### Methods

Experiment 1 was conducted between January and March of 2021 in Singapore, almost a year after the introduction of mandatory mask requirements in April, 2020. Each of 24 observers (11 females, mean age = 23.8 years, age range: 20–28 years) performed three conditions: (1) learn and test with full faces only; (2) learn and test with faces with sunglasses only; and (3) learn and test with faces with masks only. Thus, the disguise types always matched between learning and testing (i.e. congruent). None of these observers performed Experiment 2 or 3.

### Results

#### Hit rate

**Figure 2:**
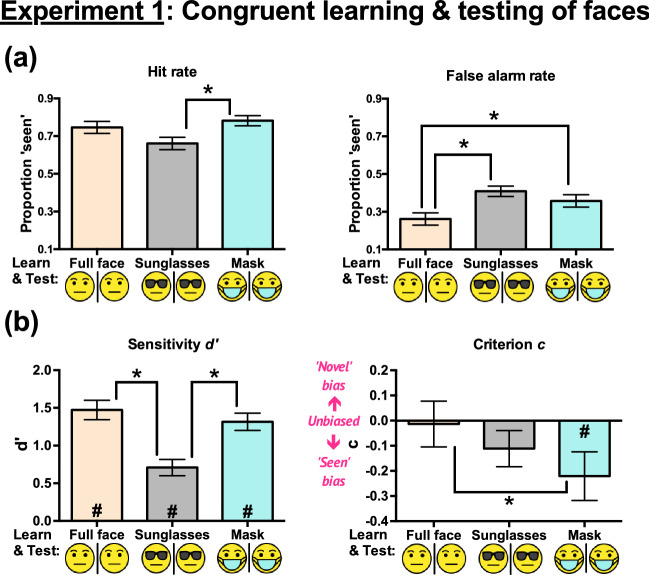
Experiment 1 results: (**a**) Hit rates and false alarm rates, and (**b**) sensitivities *d’* and criteria *c* (indicating response biases) across the three conditions. All error bars represent ± 1 SEM. The asterisks (*) indicate significant pairwise differences between conditions. The hash signs (#) in (**b**) represent *d’* or *c* significantly different from zero in the indicated conditions. The unmarked bars and comparisons were not statistically significant. The significance level was set at 0.05.

The ANOVA on hit rates (Fig. 2a) showed a significant main effect of condition, *F*(2, 46) = 7.182, *p* = 0.002, *η*_*p*_^2^ = 0.238. Mauchly’s test of sphericity indicated that the sphericity assumption was met, *χ*^2^(2) = 3.189, *p* = 0.20. Pairwise comparisons showed that full faces had no significant differences in hit rate from sunglasses (Full faces – Sunglasses = 0.085, *p* = 0.072, Cohen’s *d* = 0.454) and from masks (Masks – Full faces = 0.036, *p* = 0.25, Cohen’s *d* = 0.241). Masks led to significantly higher hit rates than sunglasses (mean difference = 0.121, *p* = 0.001, Cohen’s *d* = 0.849).

#### False alarm rate

The ANOVA on false alarm rates (Fig. 2a) showed a significant main effect of condition, *F*(2, 46) = 10.53, *p* < 0.001, *η*_*p*_^2^ = 0.314. Mauchly’s test of sphericity indicated that the sphericity assumption was met, *χ*^2^(2) = 0.075, *p* = 0.963. Pairwise comparisons showed that full faces scored significantly lower false alarm rates than sunglasses (mean difference = 0.147, *p* < 0.001, Cohen’s *d* = 0.914) and masks (mean difference = 0.095, *p* = 0.02, Cohen’s *d* = 0.588). However, the false alarm rates for sunglasses and masks were not significantly different (Sunglasses – Masks = 0.052, *p* = 0.12, Cohen’s *d* = 0.334). Overall, full faces resulted in the lowest false alarm rates.

#### Sensitivity d’

The results were also analysed in terms of sensitivity *d’* (Fig. 2b) so that both hit rates and false alarm rates were jointly considered. Separate two-tailed one-sample *t* tests showed that the *d’* values for all three conditions deviated significantly from zero (*p*s < 0.001), indicating that observers identified the studied faces in all conditions.

The ANOVA on *d’* showed a significant main effect of condition, *F*(2, 46) = 13.75, *p* < 0.001, *η*_*p*_^2^ = 0.374. Mauchly’s test of sphericity indicated that the sphericity assumption was met, *χ*^2^(2) = 0.734, *p* = 0.69. Pairwise comparisons showed that sunglasses resulted in significantly lower *d’* than full faces (mean difference = 0.764, *p* < 0.001, Cohen’s *d* = 0.992) and masks (mean difference = 0.607, *p* < 0.001, Cohen’s *d* = 0.886). However, no significant differences in *d’* were found between full faces and masks (Full faces – Masks = 0.157, *p* = 0.35, Cohen’s *d* = 0.195). Thus, only sunglasses, but not masks, led to a significantly lower *d’*.

#### Response bias (criterion c)

To detect potential response biases, we performed a separate two-tailed one-sample *t* test on criterion *c* (Fig. 2b) for each condition. Masks led to significant deviations of *c* from zero, *t*(23) = – 2.277, *p* = 0.032, Cohen’s *d* = – 0.465, indicating observers’ inclination to label any faces as seen (i.e. liberal biases). However, we did not detect significant deviations of *c* from zero for both full faces, *t*(23) = – 0.147, *p* = 0.884, Cohen’s *d* = – 0.030, and sunglasses, *t*(23) = – 1.541, *p* = 0.137, Cohen’s *d* = – 0.315. In other words, we failed to find significant biases in these two conditions.

The ANOVA on *c* showed a significant main effect of condition, *F*(2, 46) = 3.518, *p* = 0.038, *η*_*p*_^2^ = 0.133. Mauchly’s test of sphericity indicated that the sphericity assumption was met, *χ*^2^(2) = 0.366, *p* = 0.83. Pairwise comparisons showed that masks resulted in significantly more negative *c* than full faces (mean difference = 0.207, *p* = 0.037, Cohen’s *d* = 0.554). However, sunglasses did not lead to significant differences in *c* from full faces (Full faces – Sunglasses = 0.098, *p* = 0.32, Cohen’s *d* = 0.241) or from masks (Sunglasses – Masks = 0.109, *p* = 0.32, Cohen’s *d* = 0.298). Overall, only masks resulted in strong evidence of liberal bias.

## Experiment 2: learning disguised faces, testing with full faces

### Methods

Experiment 2 was conducted between April and May of 2022 in Singapore. Each of 24 observers (12 females, mean age = 21.8 years, age range: 21–24 years) performed three conditions: (1) learn and test with full faces only; (2) learn faces with sunglasses and test with full faces; and (3) learn faces with masks and test with full faces. Thus, the face memory tests always showed full faces only. None of these observers performed Experiment 1 or 3.

### Results

#### Hit rate

**Figure 3:**
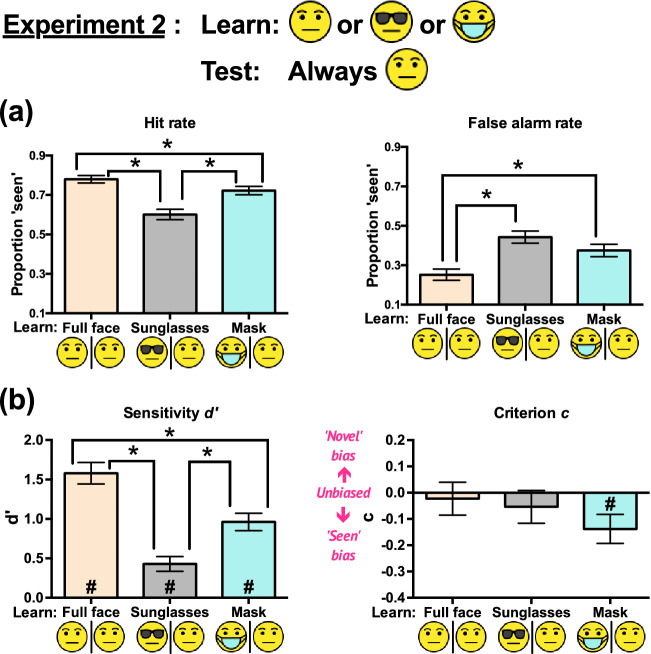
Experiment 2 results: (**a**) Hit rates and false alarm rates, and (**b**) sensitivities *d’* and criteria *c* (indicating response biases) across the three conditions. All error bars represent ± 1 SEM. The asterisks (*) indicate significant pairwise differences between conditions. The hash signs (#) in (**b**) represent *d’* or *c* significantly different from zero in the indicated conditions. The unmarked bars and comparisons were not statistically significant. The significance level was set at 0.05.

The ANOVA on hit rates (Fig. 3a) showed a significant main effect of condition, *F*(2, 46) = 15.51, *p* < 0.001, *η*_*p*_^2^ = 0.403. Mauchly’s test of sphericity indicated that the sphericity assumption was met, *χ*^2^(2) = 3.226, *p* = 0.20. All pairwise comparisons showed significant differences: Full faces – Sunglasses = 0.179, *p* < 0.001, Cohen’s *d* = 1.078; Full faces – masks = 0.058, *p* = 0.04, Cohen’s *d* = 0.445; Masks – Sunglasses = 0.121, *p* = 0.007, Cohen’s *d* = 0.667. Thus, full faces scored the highest hit rates, followed by masks, and sunglasses scored the lowest.

#### False alarm rate

The ANOVA on false alarm rates (Fig. 3a) showed a significant main effect of condition, *F*(2, 46) = 16.78, *p* < 0.001, *η*_*p*_^2^ = 0.422. Mauchly’s test of sphericity indicated that the sphericity assumption was met, *χ*^2^(2) = 1.169, *p* = 0.56. Pairwise comparisons showed that full faces scored significantly lower false alarm rates than sunglasses (mean difference = 0.190, *p* < 0.001, Cohen’s *d* = 1.311) and masks (mean difference = 0.123, *p* = 0.005, Cohen’s *d* = 0.693). However, the false alarm rates for sunglasses and masks were not significantly different (Sunglasses – Masks = 0.067, *p* = 0.06, Cohen’s *d* = 0.408). Overall, full faces resulted in the lowest false alarm rates.

#### Sensitivity d’

Separate two-tailed one-sample *t* tests showed that the *d’* values for all three conditions (Fig. 3b) deviated significantly from zero (*p*s < 0.001), indicating that observers identified the studied faces in all conditions.

The ANOVA on *d’* showed a significant main effect of condition, *F*(2, 46) = 27.41, *p* < 0.001, *η*_*p*_^2^ = 0.544. Mauchly’s test of sphericity indicated that the sphericity assumption was met, *χ*^2^(2) = 1.47, *p* = 0.48. All pairwise comparisons showed significant differences: Full faces – Sunglasses = 1.15, *p* < 0.001, Cohen’s *d* = 1.646; Full faces – masks = 0.618, *p* = 0.003, Cohen’s *d* = 0.725; Masks – Sunglasses = 0.532, *p* = 0.003, Cohen’s *d* = 0.734. Thus, full faces scored the highest *d’*, followed by masks, and sunglasses scored the lowest.

#### Response bias (criterion c)

Separate two-tailed one-sample *t* tests on criterion *c* (Fig. 3b) showed significant deviations from zero for masks only, *t*(23) = – 2.50, *p* = 0.02, Cohen’s *d* = – 0.51, but no significant deviations for full faces, *t*(23) = – 0.37, *p* = 0.72, Cohen’s *d* = – 0.075, and for sunglasses, *t*(23) = – 0.87, *p* = 0.40, Cohen’s *d* = – 0.177.

The ANOVA on *c* did not show any significant main effect of condition, *F*(2, 46) = 1.43, *p* = 0.25, *η*_*p*_^2^ = 0.058. Mauchly’s test of sphericity indicated that the sphericity assumption was met, *χ*^2^(2) = 1.19, *p* = 0.55.

## Experiment 3: learning full faces, testing with disguised faces

### Methods

Experiment 3 was conducted between May and June of 2022 in Singapore. Each of 24 observers (9 females, mean age = 24.7 years, age range: 21–41 years) performed three conditions: (1) learn and test with full faces only; (2) learn full faces and test with faces with sunglasses; and (3) learn full faces and test with faces with masks. Thus, the face learning sessions always showed full faces only. None of these observers performed Experiment 1 or 2.

### Results

#### Hit rate

**Figure 4:**
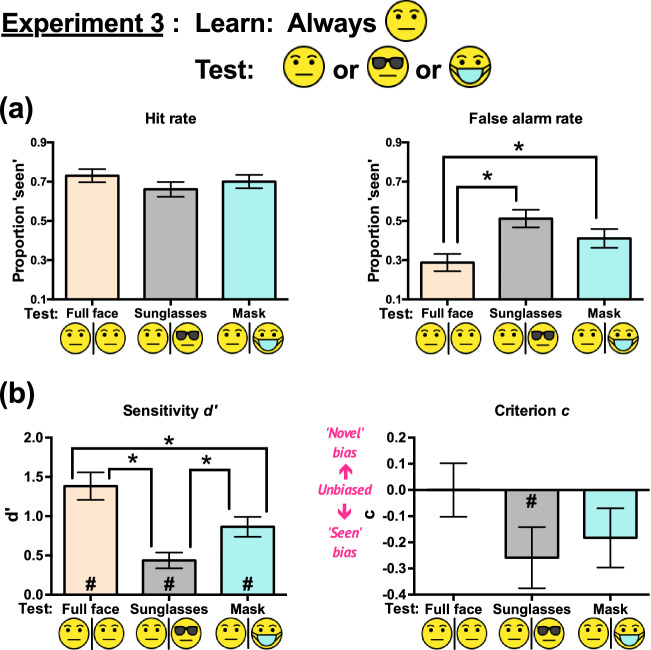
Experiment 3 results: (**a**) Hit rates and false alarm rates, and (**b**) sensitivities *d’* and criteria *c* (indicating response biases) across the three conditions. All error bars represent ± 1 SEM. The asterisks (*) indicate significant pairwise differences between conditions. The hash signs (#) in (**b**) represent *d’* or *c* significantly different from zero in the indicated conditions. The unmarked bars and comparisons were not statistically significant. The significance level was set at 0.05.

The ANOVA on hit rates (Fig. 4a) did not show a significant main effect of condition, *F*(2, 46) = 1.66, *p* = 0.20, *η*_*p*_^2^ = 0.067. Mauchly’s test of sphericity indicated that the sphericity assumption was met, *χ*^2^(2) = 5.27, *p* = 0.07.

#### False alarm rate

The ANOVA on false alarm rates (Fig. 4a) showed a significant main effect of condition, *F*(1.571, 36.132) = 11.82, *p* < 0.001, *η*_*p*_^2^ = 0.340. Mauchly’s test of sphericity indicated that the sphericity assumption was violated, *χ*^2^(2) = 7.02, *p* = 0.03; thus, Greenhouse–Geisser correction was applied. Pairwise comparisons showed that full faces scored significantly lower false alarm rates than sunglasses (mean difference = 0.224, *p* < 0.001, Cohen’s *d* = 1.003) and masks (mean difference = 0.123, *p* = 0.002, Cohen’s *d* = 0.550). However, the false alarm rates for sunglasses and masks were not significantly different (mean difference = 0.101, *p* = 0.06, Cohen’s *d* = 0.453). Overall, full faces resulted in the lowest false alarm rates.

#### Sensitivity d’

Separate two-tailed one-sample *t* tests showed that the *d’* values for all three conditions (Fig. 4b) deviated significantly from zero (*p*s < 0.001), indicating that observers identified the studied faces in all conditions.

The ANOVA on *d’* showed a significant main effect of condition, *F*(2, 46) = 19.11, *p* < 0.001, *η*_*p*_^2^ = 0.454. Mauchly’s test of sphericity indicated that the sphericity assumption was met, *χ*^2^(2) = 0.252, *p* = 0.88. All pairwise comparisons showed significant differences: Full faces – Sunglasses = 0.947, *p* < 0.001, Cohen’s *d* = 1.409; Full faces – Masks = 0.519, *p* = 0.007, Cohen’s *d* = 0.772; Masks – Sunglasses = 0.428, *p* = 0.007, Cohen’s *d* = 0.637. Thus, full faces scored the highest *d’*, followed by masks, and sunglasses scored the lowest.

#### Response bias (criterion c)

Separate two-tailed one-sample *t* tests on criterion *c* (Fig. 4b) showed significant deviations from zero for sunglasses only, *t*(23) = – 2.21, *p* = 0.037, Cohen’s *d* = – 0.45, but no significant deviations for full faces, *t*(23) = 0.001, *p* = 0.999, Cohen’s *d* = 0.0002, and for masks, *t*(23) = – 1.61, *p* = 0.12, Cohen’s *d* = – 0.33.

The ANOVA on *c* did not show any significant main effect of condition, *F*(1.479, 34.017) = 2.33, *p* = 0.12, *η*_*p*_^2^ = 0.092. Mauchly’s test of sphericity indicated that the sphericity assumption was violated, *χ*^2^(2) = 9.55, *p* = 0.008; thus, Greenhouse–Geisser correction was applied.

## Comparing results across three experiments

First, we investigated whether the results for the full face condition were consistent across experiments. Then, we compared the effects of masks and sunglasses on different experiments. The comparisons were performed in terms of *d’* and *c*.

### Full face conditions in the three experiments

Both one-way ANOVAs on *d’* and *c* separately showed no significant differences across different groups of observers learning and testing with full faces in the three experiments (For *d’*, *F*(2, 69) = 0.435, *p* = 0.65, *η*_*p*_^2^ = 0.012; for *c*, *F*(2, 69) = 0.018, *p* = 0.98, *η*_*p*_^2^ = 0.0005).

### Disguise effects

**Figure 5:**
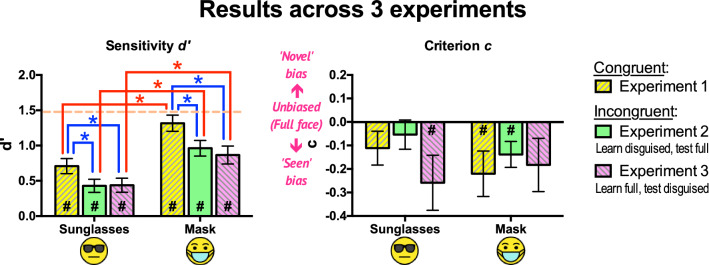
Sensitivities *d’* and criteria *c* (indicating response biases) across the three experiments. Sensitivities: Incongruent disguises (Experiments 2 & 3) led to significantly lower *d’* than congruent disguises (Experiment 1), and sunglasses led to significantly lower *d’* than masks, as indicated by the asterisks (*). Broken line (in orange): *d’* for presenting only full faces, averaged across all three experiments. Criteria: No significant effects were found between sunglasses and masks, and among congruent and incongruent conditions. Presenting only full faces resulted in no significant bias (mean *c* = – 0.01, *p* = 0.81; not drawn). All error bars represent ± 1 SEM. The hash signs (#) represent significant differences from zero in the indicated conditions. The unmarked bars and comparisons were not statistically significant. The significance level was set at 0.05.

Separate two-way mixed ANOVAs (3 Experiments × 2 Disguises: Sunglasses, Mask) were performed on *d’* and *c* (Fig. 5).

#### Sensitivity d’

We found significant main effects of both Experiment, *F*(2, 69) = 5.75, *p* = 0.005, *η*_*p*_^2^ = 0.143, and Disguise, *F*(1, 69) = 39.25, *p* < 0.001, *η*_*p*_^2^ = 0.363. There was no significant interaction, *F*(2, 69) = 0.387, *p* = 0.68, *η*_*p*_^2^ = 0.011. This again indicated significantly lower *d’* with sunglasses than with masks consistently across experiments (average difference = 0.523, Cohen’s *d* = 0.976).

Post hoc between-subjects pairwise comparisons (with Tukey corrections) showed that congruent disguises across memory test stages in Experiment 1 led to significantly higher *d’* than incongruent disguises in Experiments 2 and 3 did (Experiment 1 – Experiment 2 = 0.317, *p* = 0.022, Cohen’s *d* = 0.591; Experiment 1 – Experiment 3 = 0.361, *p* = 0.008, Cohen’s *d* = 0.673). There were no significant differences in *d’* between Experiments 2 and 3 (mean difference = 0.044, *p* = 0.92, Cohen’s *d* = 0.082).

#### Response bias (criterion c)

There were no significant main effects of Experiment, *F*(2, 69) = 0.71, *p* = 0.50, *η*_*p*_^2^ = 0.02, Disguise, *F*(1, 69) = 0.46, *p* = 0.50, *η*_*p*_^2^ = 0.007, or interaction, *F*(2, 69) = 1.01, *p* = 0.37, *η*_*p*_^2^ = 0.029. In other words, we could not find any significant effects of disguise types or congruence on response bias.

## Discussion

The present study investigated the effects of face masks and sunglasses on face identification in three face memory experiments, which were designed to compare also the effects of congruent (full face/full face, or disguised face/disguised face) and incongruent face types (full face/disguised face, or disguised face/full face) across the encoding and retrieval stages.

To summarize, we found that both face masks and sunglasses impaired face identification but in different manners: (1) Sunglasses led to stronger impairment than face masks did (lowering sensitivity by 50% on average). (2) Face masks reduced sensitivity only when disguises were incongruent between encoding and retrieval (Experiments 2 and 3). (3) Congruently presenting face masks (Experiment 1) led to a liberal bias favouring “seen” responses. (4) Responses were unbiased with full faces in both learning and testing, although there were some tendencies of liberal biases with disguised faces. (5) For both sunglasses and face masks, sensitivity was on average 35% lower for incongruent encoding and retrieval than congruent ones.

### Different effects of upper and lower occlusions

The greater impairment with sunglasses supports the notion that the eye region contains major diagnostic information for processing face identity, as implicated by a range of studies. For example, monkey neurophysiology showed that face sensitive cells were particularly sensitive to the eyes^24,25^. Humans often direct their eye movements to (or near) the eye region during face recognition^26,27^, especially in the initial fixations^28–30^. Individuals who made effective use of the visual information from the eye region often showed better face memory or face recognition ability^31–33^. Consistent with many previous studies showing that occluding the eye region impairs face identification, we additionally found that the impairment was present regardless of congruence in disguise between encoding and retrieval (though with different strengths; *cf*^19^). Sensitivity to diagnostic eye information probably starts from the early stages of face identity processing, as suggested in behavioural^14,34^ and electrophysiological findings^15,35,36^.

In contrast, face masks consistently led to weaker impairment than sunglasses in sensitivity to face identification. Despite that, such reduced impairment was still significant in Experiments 2 and 3 (*cf*^1,4–6^). This suggests that the lower region of the face might be less diagnostic than the eye region, even though the lower region contains multiple features including the mouth, the chin, and a large part of the nose. Our results are broadly consistent with many previous findings, which show that occluding lower facial regions has smaller effects than occluding upper facial regions does^7,12,20^. Nevertheless, the present results did not agree with those from two recent studies^21,22^ comparing the effects of sunglasses and face masks (whereas these studies found different results from each other; see “Introduction”). It should be noted that these two studies required simultaneous matching between two faces, while the present study used a face memory test design. It is possible that the task demands may interact with the effects of different disguises on face identity processing.

### Support for the encoding specificity principle

Our results provide direct evidence for the encoding specificity principle in face identity processing, as incongruent occlusions (Experiments 2 and 3) led to significantly lower sensitivities than congruent occlusions did (Experiment 1). In fact, congruently learning and testing with face masks did not even reduce sensitivity from learning and testing of full faces in Experiment 1. The difficulty to associate disguised faces with their corresponding full faces (in Experiments 2 and 3) may be a result of the holistic processing of faces (e.g.^37–44^), where multiple facial features are integrated to form a perceptual whole in the face representation. Holistic processing may be illustrated by the “composite face”^45^, where the top half of an individual’s face is aligned to the bottom half of another individual’s face. Even though the top half remains the same, replacing the bottom half by another identity introduces a visual illusion (composite face illusion) that the top half appears different too (see ref.^42^ for review). In our context, the unoccluded part of the disguised face might not have undergone the same perceptual transformations that holistic processing has done on the full face. As a result, the same facial features in the disguised face and the corresponding full face might be perceived differently.

Recently, Hsiao et al.^3^ also found lower sensitivities from their incongruent conditions (unmasked face/masked face, masked face/unmasked face) than their unmasked face/unmasked face condition (see also ref.^46^). The incongruence effect may be partly attributed to the cost of adjusting the eye movement patterns to accommodate the mismatch, as identification of masked faces may benefit from focusing the gaze on the eye region. Interestingly, sensitivity reduction was observed even among their congruent conditions (i.e. masked face/masked face < unmasked face/unmasked face). This may be due to the absence of head and hair outlines in their cropped face stimuli such that fewer facial features (probably just the eyes) were visible for masked-face identification. In contrast, our study did not show such sensitivity reduction with uncropped face stimuli showing outer facial features.

### Higher false alarm rates with disguised faces

Notably, both sunglasses and face masks led to significantly higher false alarm rates than presenting only full faces in all three experiments. In addition to generally lowering sensitivity, the higher false alarm rates appeared to contribute to tendencies of liberal biases in both disguised conditions. In contrast, when full faces were presented during both encoding and retrieval, face identification remained unbiased. These findings suggest that occluding part of the face may affect the decision process leading to criterion shifts (e.g.^47,48^), perhaps through reducing the general familiarity of the stimulus class of faces^49^. Considering the general familiarity account is especially interesting, as one could have argued that masked faces might have already become familiar from prolonged natural exposure due to extensive, mandatory mask-wearing requirements. However, recent results gathered during the COVID-19 pandemic suggest that familiarity of masked faces still cannot match familiarity of unoccluded faces arising from lifelong experience, as challenges in recognizing masked faces continue to persist despite more than 1 year of widespread exposure^2,22^. In addition, the poorer masked-face memory reported by Freud et al.^1,2^ may not always reflect reduced sensitivity, but rather criterion shifts in the decision process especially during congruent encoding and retrieval, considering that the CFMT scores they used did not distinguish between sensitivity and criterion as we did. Nevertheless, it should be noted that not all disguised conditions in our study introduced significant liberal biases, probably due to mostly small or non-significant differences in hit rate among disguised face and full face conditions.

### Limitations

One potential limitation of the study arose from using only one face photo per individual (the database did not provide a second photo), which could allow for matching of low-level visual patterns^50^ rather than matching for high-level face identity information. To partly address this issue, the size of the face was varied randomly across presentations. This reduces the reliance of low-level processing^51^, while the processing of face identity remains robust across changes in face size (e.g.^52–54^). Despite that, future studies using multiple face photos per individual would be desirable.

## Conclusion

The present study demonstrates that occlusions of face parts can affect face identification in multiple ways. Future studies may focus on the specific effects of different disguise types under various encoding/retrieval conditions. Findings of such studies may contribute to understanding of the holistic processing of faces and may lead to practical implications, for example, in the training of disguised face recognition and in the validity of eyewitness testimony as disguises have become more common.

## Data Availability

The datasets generated during and/or analysed during the current study are available from the corresponding author on reasonable request.

## References

[1] Freud E, Stajduhar A, Rosenbaum RS, Avidan G, Ganel T (2020). The COVID-19 pandemic masks the way people perceive faces. Sci. Rep..

[2] Freud E (2022). Recognition of masked faces in the era of the pandemic: No improvement despite extensive natural exposure. Psychol. Sci..

[3] Hsiao JH-W, Liao W, Tso RVY (2022). Impact of mask use on face recognition: An eye-tracking study. Cogn. Res. Princ. Implic..

[4] Marini M, Ansani A, Paglieri F, Caruana F, Viola M (2021). The impact of facemasks on emotion recognition, trust attribution and re-identification. Sci. Rep..

[5] Carragher DJ, Hancock PJB (2020). Surgical face masks impair human face matching performance for familiar and unfamiliar faces. Cogn. Res. Princ. Implic..

[6] Estudillo AJ, Hills P, Wong HK (2021). The effect of face masks on forensic face matching: An individual differences study. J. Appl. Res. Mem. Cogn..

[7] McKelvie SJ (1976). The role of eyes and mouth in the memory. Am. J. Psychol..

[8] Tulving E, Thomson DM (1973). Encoding specificity and retrieval processes in episodic memory. Psychol. Rev..

[9] Shapiro PN, Penrod S (1986). Meta-analysis of facial identification studies. Psychol. Bull..

[10] Carlson CA (2021). Testing encoding specificity and the diagnostic feature-detection theory of eyewitness identification, with implications for showups, lineups, and partially disguised perpetrators. Cogn. Res. Princ. Implic..

[11] Lim DY, Lee ALF, Or CC-F (2022). Incongruence in lighting impairs face identification. Front. Psychol..

[12] Roberts T, Bruce V (1988). Feature saliency in judging the sex and familiarity of faces. Perception.

[13] Manley KD, Chan JCK, Wells GL (2019). Do masked-face lineups facilitate eyewitness identification of a masked individual?. J. Exp. Psychol..

[14] Vinette C, Gosselin F, Schyns PG (2004). Spatio-temporal dynamics of face recognition in a flash: It’s in the eyes. Cogn. Sci..

[15] Itier RJ, Alain C, Sedore K, McIntosh AR (2007). Early face processing specificity: It’s in the eyes!. J. Cogn. Neurosci..

[16] Sadr J, Jarudi I, Sinha P (2003). The role of eyebrows in face recognition. Perception.

[17] Graham DL, Ritchie KL (2019). Making a spectacle of yourself: The effect of glasses and sunglasses on face perception. Perception.

[18] Mansour JK (2020). Impact of disguise on identification decisions and confidence with simultaneous and sequential lineups. Law Hum. Behav..

[19] Hockley WE, Hemsworth DH, Consoli A (1999). Shades of the mirror effect: Recognition of faces with and without sunglasses. Mem. Cogn..

[20] Nguyen TB, Pezdek K (2017). Memory for disguised same- and cross-race faces: The eyes have it. Vis. Cogn..

[21] Noyes E, Davis JP, Petrov N, Gray KLH, Ritchie KL (2021). The effect of face masks and sunglasses on identity and expression recognition with super-recognizers and typical observers. R. Soc. Open Sci..

[22] Bennetts RJ, Humphrey PJ, Zielinska P, Bate S (2022). Face masks versus sunglasses: Limited effects of time and individual differences in the ability to judge facial identity and social traits. Cogn. Res. Princ. Implic..

[23] Macmillan NA, Creelman CD (2005). Detection Theory: A User’s Guide.

[24] Freiwald WA, Tsao DY, Livingstone MS (2009). A face feature space in the macaque temporal lobe. Nat. Neurosci..

[25] Issa EB, DiCarlo JJ (2012). Precedence of the eye region in neural processing of faces. J. Neurosci. Res..

[26] Henderson JM, Williams CC, Falk RJ (2005). Eye movements are functional during face learning. Mem. Cogn..

[27] Barton JJ, Radcliffe N, Cherkasova MV, Edelman J, Intriligator JM (2006). Information processing during face recognition: The effects of familiarity, inversion, and morphing on scanning fixations. Perception.

[28] Hsiao JH-W, Cottrell G (2008). Two fixations suffice in face recognition. Psychol. Sci..

[29] Peterson MF, Eckstein MP (2012). Looking just below the eyes is optimal across face recognition tasks. Proc. Natl. Acad. Sci. U. S. A..

[30] Or CC-F, Peterson MF, Eckstein MP (2015). Initial eye movements during face identification are optimal and similar across cultures. J. Vis..

[31] Hills PJ, Ross DA, Lewis MB (2011). Attention misplaced: The role of diagnostic features in the face-inversion effect. J. Exp. Psychol..

[32] Sekiguchi T (2011). Individual differences in face memory and eye fixation patterns during face learning. Acta Psychol..

[33] Royer J (2018). Greater reliance on the eye region predicts better face recognition ability. Cognition.

[34] Schyns PG, Bonnar L, Gosselin F (2002). Show me the features! Understanding recognition from the use of visual information. Psychol. Sci..

[35] Nemrodov D, Itier RJ (2011). The role of eyes in early face processing: A rapid adaptation study of the inversion effect. Br. J. Psychol..

[36] Nemrodov D, Anderson T, Preston FF, Itier RJ (2014). Early sensitivity for eyes within faces: A new neuronal account of holistic and featural processing. Neuroimage.

[37] Farah MJ, Wilson KD, Drain M, Tanaka JN (1998). What is “special” about face perception?. Psychol. Rev..

[38] Maurer D, Le Grand R, Mondloch CJ (2002). The many faces of configural processing. Trends Cogn. Sci..

[39] McKone E, Martini P, Nakayama K, Peterson MA, Rhodes G (2003). Isolating holistic processing in faces (and perhaps objects). Perception of Faces, Objects, and Scenes: Analytic and Holistic Processes.

[40] Rossion B (2008). Picture-plane inversion leads to qualitative changes of face perception. Acta Psychol..

[41] Rossion B (2009). Distinguishing the cause and consequence of face inversion: The perceptual field hypothesis. Acta Psychol..

[42] Rossion B (2013). The composite face illusion: A whole window into our understanding of holistic face perception. Vis. Cogn..

[43] Tanaka JW, Farah MJ (1993). Parts and wholes in face recognition. Q. J. Exp. Psychol..

[44] Tanaka JW, Farah MJ, Peterson MA, Rhodes G (2003). The holistic representation of faces. Perception of Faces, Objects, and Scenes: Analytic and Holistic Processes.

[45] Young AW, Hellawell D, Hay DC (1987). Configurational information in face perception. Perception.

[46] Garcia-Marques T, Oliveira M, Nunes L (2022). That person is now with or without a mask: How encoding context modulates identity recognition. Cogn. Res. Princ. Implic..

[47] Miller MB, Wolford GL (1999). Theoretical commentary: The role of criterion shift in false memory. Psychol. Rev..

[48] Verde MF, Rotello CM (2007). Memory strength and the decision process in recognition memory. Mem. Cogn..

[49] Vokey JR, Hockley WE (2012). Unmasking a shady mirror effect: Recognition of normal versus obscured faces. Q. J. Exp. Psychol..

[50] Hancock PJB, Bruce V, Burton MA (2000). Recognition of unfamiliar faces. Trends Cogn. Sci..

[51] Webster MA, MacLeod DIA (2011). Visual adaptation and face perception. Philos. Trans. R. Soc. B.

[52] Jeffery L, Rhodes G, Busey T (2006). View-specific coding of face shape. Psychol. Sci..

[53] Lee Y, Matsumiya K, Wilson HR (2006). Size-invariant but viewpoint-dependent representation of faces. Vis. Res..

[54] Yamashita JA, Hardy JL, De Valois KK, Webster MA (2005). Stimulus selectivity of figural aftereffects for faces. J. Exp. Psychol..

